# Upper Pole Nephrectomy: A Simplified Technique Using a Retroperitoneal Laparoscopic Approach

**DOI:** 10.1155/2011/570790

**Published:** 2011-12-08

**Authors:** Joana Pereira, Angélica Osório, João Moreira-Pinto, José Cidade-Rodrigues, Carlos Enes, Armando Reis, João Ribeiro-Castro

**Affiliations:** Unidade Maria Pia, Centro Hospitalar do Porto, Rua da Boavista, No. 827, 4050-111 Porto, Portugal

## Abstract

*Objective*. To describe a simplified technique already used in our institution for several years in the open heminephrectomy for duplication anomalies, now performed through a retroperitoneal laparoscopic approach. *Methods*. The technique begins with upper pole parenchyma incision since the demarcation between the affected upper moiety and the healthy lower pole is easily established. The dissection proceeds until the urothelium of the collecting system is entered, which will guide further excision, minimizing damage of the surrounding structures. The vascular supply is then identified since the upper pole is attached to the remaining renal parenchyma only by these structures that can be safely divided. Dissection and division of the ectopic ureter is carried next. *Results*. The operative time was 188 minutes. The blood loss was not significant, and there were no other complications during the procedure. The patient was discharged home 48 hours after the procedure, without any early or late postoperative complications. *Conclusion*. We believe this simplified technique allows a safer excision of nonfunctioning upper pole renal tissue by avoiding the initial dissection of the renal hilum, which associated with the known advantages of a laparoscopic approach makes us consider it the procedure of choice for upper pole nephrectomy in children.

## 1. Introduction

Upper pole nephrectomy is a treatment option when duplication anomalies are associated with ureteral ectopia or ureterocele, and an associated nonfunctioning upper pole moiety [1].

Jordan and Winslow were the first to describe a transperitoneal laparoscopic partial nephrectomy in children [2]. The retroperitoneal approach has been privileged by nearly all pediatric urologists for conventional surgery [3]. Moreover, after Gaur developed the balloon dissection technique and reported the first laparoscopic retroperitoneal nephrectomy in a flank position in 1994 [4], the popularity of retroperitoneoscopy increased. In 2000, Miyazato et al. was the first to report a partial nephrectomy via a retroperitoneal approach in a pediatric patient [5], and there are now several series that demonstrate the safety and efficacy of laparoscopic approaches for benign renal disease in infants and children. 

Laparoscopy provides an excellent overview of the anatomical structures, with magnification allowing exact pole ablation along the anatomical border [6–8]. Postoperative pain is reduced, as are wound complications, scaring is minimal and there is a shorter hospital stay and an earlier return to normal activities. When compared with transperitoneal access, the retroperitoneal approach appears to have several advantages; it improves the ease of direct kidney access by developing the existing potential retroperitoneal space, and reduces the risk of injury and interference from intraabdominal organs and great vessels [7]. Further, the risk of postoperative bowel adhesion formation is avoided and previous abdominal surgeries are not a contraindication [3, 7].

The standard heminephrectomy techniques involve initial hilar dissection to isolate and divide the upper pole blood supply, which may at times be a cumbersome process with an inherent risk of injury to the lower pole vascular pedicle [1]. Jednak et al. described a simplified technique of upper pole heminephrectomy in which the initial dissection of vascular pedicle is avoided [1], and after using that same technique in open surgery for several years at our institution, we now describe it as being performed through a retroperitoneal laparoscopic approach.

## 2. Materials and Methods

The surgery was performed on a three-year-old girl with a right duplicated system. She had a hydronephrotic moiety and repeated urinary tract infections. The ectopic ureter corresponded to a nonfunctioning upper renal pole, as shown by renal scintigraphy using mercaptoacetyltriglycine (MAG3). The voiding cystourethrography did not show ureterovesical reflux. Prior to surgery, a cystourethroscopy identifying the ectopic orifice at the level of the bladder neck was performed.

The patient was placed in a lateral decubitus position, the pressure points were carefully padded, and a roll was placed under the pelvis to elevate the affected side. A transverse 5 mm skin incision was made at the lower border of the tip of the twelfth rib, and the retroperitoneum was approached by a muscle-splitting blunt dissection. Following development of the retroperitoneal space with a dissection balloon as described by Gaur, a 5 mm Hasson trocar was inserted and the retroperitoneum was insufflated with carbon dioxide to a maximum pressure of 10–12 mmHg. The second and third trocars were placed under direct vision in the anterior and posterior axillary line, and a finger width from the top of the iliac crest. Gerota's fascia was incised and the posterior surface of the kidney dissected using a combination of sharp and blunt dissection, which allowed identification of the adrenal gland and both renal moieties, dysplastic and healthy. The ectopic ureter was traced above and below the renal hilum and isolated from the healthy one, taking care to preserve the structure and irrigation of the last. The renal hilum was approached, but no ligations were performed at this moment since it is hard to identify which vessels supply each pole and an erroneous ligation could compromise areas of unaffected parenchyma or the lower pole ureter. The first step was upper pole parenchyma incision because demarcation between the affected upper moiety and the healthy lower one was easily established. A harmonic scalpel was used to divide the parenchyma, and the dissection proceeded until the urothelium of the collecting system was entered, which guided further excision and minimized damage of the surrounding structures. After parenchyma incision, the branches of the renal artery and vein supplying the upper pole could be easily identified, since they and the ureter were the only structures attaching the ablated tissue to the remaining renal parenchyma. Endoscopic clips were used to ligate the vessels separately. Finally, the ectopic ureter was pulled by the site where it was approached at the beginning of the surgery and it was ligated with an endoloop and divided as distal as possible. The resected pole and ureter were placed in an endobag and retrieved directly through a port incision. A drain was left indwelling in the perirenal space, and all port sites were carefully closed.

## 3. Results

The operative time was 188 minutes, and there were no complications during the procedure. The estimated blood loss was less than 30 cc, and the postoperative hemoglobin was 10 gm/dL. Oral diet and ambulation were resumed on the first postoperative day, and the patient was discharged home on the second post-operative day. There were no late complications, and the aesthetic result was excellent.

## 4. Discussion

Laparoscopic heminephrectomy in duplex kidneys is more difficult than nephrectomy due to the increased risk of haemorrhage, urine leak, and vascular compromise of the remaining renal moiety [6, 9]. The general principles of the standard technique include the identification and division of the hilar vessels and distal ureter, followed by separation of the affected pole from the remainder of the kidney [10].

Overall procedure success depends on the maximal preservation of lower pole function, which requires careful identification of the lower pole vessels to prevent inadvertent injury, minimal excision of healthy lower pole parenchyma, and identification and protection of the lower pole ureter during upper ureteral dissection [1]. The initial dissection of the hilar vessels is not always easy, and an inadvertent ligation of lower pole vessels might result in lower pole ischemia and hypertension, or even total renal loss [11]. 

In this simplified technique, and since the demarcation between the affected upper pole and lower healthy pole can easily be done, the collecting system is initially entered and used to guide excision. This reduces the risk of inadvertent entry into the lower pole collecting system and healthy lower pole tissue excision [1].

The identification of the upper pole vessels and ureter is safer after this maneuver, since they are the only structures attaching the excised pole to the remaining parenchyma. Therefore, nondissection of the hilum prevents iatrogenic lesions, reduces surgical time and prevents the ligation of branches that irrigate the lower pole, thus avoiding decreased function in this segment [12].

The main disadvantage of retroperitoneoscopy is the small working space, especially in younger children, where complications seem more frequent and agedependent [13, 14]. In children less than 2 years of age, the transperitoneal approach reduces the risk of conversion and of severe complications [14].

We believe this simplified technique allows a safer excision of nonfunctioning upper pole renal tissue by avoiding the initial dissection of renal hilum, which associated with the already described advantages of a laparoscopic approach makes us consider it the procedure of choice for upper pole nephrectomy in children.
